# Genetic Associations and Differential mRNA Expression Levels of Host Genes Suggest a Viral Trigger for Endemic Pemphigus Foliaceus

**DOI:** 10.3390/v14050879

**Published:** 2022-04-23

**Authors:** Valéria Bumiller-Bini Hoch, Ana Flávia Kohler, Danillo G. Augusto, Sara Cristina Lobo-Alves, Danielle Malheiros, Gabriel Adelman Cipolla, Angelica Beate Winter Boldt, Karin Braun-Prado, Michael Wittig, Andre Franke, Claudia Pföhler, Margitta Worm, Nina van Beek, Matthias Goebeler, Miklós Sárdy, Saleh Ibrahim, Hauke Busch, Enno Schmidt, Jennifer Elisabeth Hundt, Patrícia Savio de Araujo-Souza, Maria Luiza Petzl-Erler

**Affiliations:** 1 Laboratory of Human Molecular Genetics, Department of Genetics, Federal University of Paraná (UFPR), Curitiba 81531-980, Brazil; valeriabumiller@gmail.com (V.B.-B.H.); danillo@augusto.bio.br (D.G.A.); saralobo5@yahoo.com.br (S.C.L.-A.); dani_malheiros@ufpr.br (D.M.); gabriel.cipolla@ufpr.br (G.A.C.); angelicaboldt@gmail.com (A.B.W.B.); kbraun@ufpr.br (K.B.-P.); 2 Postgraduate Program in Genetics, Department of Genetics, Federal University of Paraná (UFPR), Curitiba 81531-980, Brazil; anaflaviakohler@gmail.com; 3 Research Institut Pelé Pequeno Príncipe, Curitiba 80250-060, Brazil; 4 Institute of Clinical Molecular Biology (IKMB), Christian-Albrechts-University of Kiel, 24105 Kiel, Germany; m.wittig@mucosa.de (M.W.); a.franke@mucosa.de (A.F.); 5 Saarland University Medical Center, Department of Dermatology, 66421 Homburg, Germany; claudia.pfoehler@uks.eu; 6 Division of Allergy and Immunology, Department of Dermatology, Venerology and Allergy, Charité-Universitätsmedizin Berlin, 10117 Berlin, Germany; margitta.worm@charite.de; 7 Department of Dermatology, University of Lübeck, 23562 Lübeck, Germany; nina.vanbeek@uksh.de (N.v.B.); enno.schmidt@uksh.de (E.S.); 8 Department of Dermatology, Venereology and Allergology, University Hospital Würzburg, 97080 Würzburg, Germany; goebeler_m1@ukw.de; 9 Department of Dermatology and Allergy, University Hospital, LMU Munich, 80539 Munich, Germany; titkarsag.bor@med.semmelweis-univ.hu; 10 Department of Dermatology, Venereology and Dermatooncology, Semmelweis University, 1085 Budapest, Hungary; 11 Sharjah Institute for Medical Research, University of Sharjah, Sharjah 27272, United Arab Emirates; saleh.ibrahim@uksh.de; 12 Lübeck Institute of Experimental Dermatology (LIED), University of Lübeck, 23562 Lübeck, Germany; hauke.busch@uni-luebeck.de (H.B.); jennifer.hundt@uni-luebeck.de (J.E.H.); 13 Laboratory of Immunogenetics and Histocompatibility, Department of Genetics, Federal University of Paraná, Curitiba 81531-980, Brazil; psas@ufpr.br

**Keywords:** endemic pemphigus foliaceus, virus, genetic association, differential gene expression, autoimmune disease, environmental factors

## Abstract

The long search for the environmental trigger of the endemic pemphigus foliaceus (EPF, fogo selvagem) has not yet resulted in any tangible findings. Here, we searched for genetic associations and the differential expression of host genes involved in early viral infections and innate antiviral defense. Genetic variants could alter the structure, expression sites, or levels of the gene products, impacting their functions. By analyzing 3063 variants of 166 candidate genes in 227 EPF patients and 194 controls, we found 12 variants within 11 genes associated with differential susceptibility (*p* < 0.005) to EPF. The products of genes *TRIM5*, *TPCN2*, *EIF4E*, *EIF4E3*, *NUP37*, *NUP50*, *NUP88*, *TPR*, *USP15*, *IRF8*, and *JAK1* are involved in different mechanisms of viral control, for example, the regulation of viral entry into the host cell or recognition of viral nucleic acids and proteins. Only two of nine variants were also associated in an independent German cohort of sporadic PF (75 patients, 150 controls), aligning with our hypothesis that antiviral host genes play a major role in EPF due to a specific virus–human interaction in the endemic region. Moreover, *CCL5*, *P4HB*, and *APOBEC3G* mRNA levels were increased (*p* < 0.001) in CD4+ T lymphocytes of EPF patients. Because there is limited or no evidence that these genes are involved in autoimmunity, their crucial role in antiviral responses and the associations that we observed support the hypothesis of a viral trigger for EPF, presumably a still unnoticed flavivirus. This work opens new frontiers in searching for the trigger of EPF, with the potential to advance translational research that aims for disease prevention and treatment.

## 1. Introduction

Pemphigus is a broad term denoting a group of potentially life-threatening autoimmune blistering skin diseases. The major clinical forms are pemphigus vulgaris (PV) and pemphigus foliaceus (PF). Pemphigus manifests as flaccid blisters and erosions erupting on the skin (PF and PV) and oral mucosa (PV only). The hallmark of pemphigus is the presence of autoantibodies against the desmosomal cadherins desmoglein 3 (DSG3, in PV) and desmoglein 1 (DSG1, in PF and patients with mucocutaneous PV) on the cell surface of neighboring epidermal keratinocytes. Desmosomes are essential intercellular adhering junctions in keratinocytes. The IgG anti-desmoglein antibodies are critical players in the loss of intercellular connections (acantholysis), resulting from the desmosomes’ breakdown [1]. 

PF is less common worldwide than PV [2,3]. However, an endemic form of PF (EPF), also known as *fogo selvagem* (meaning wild fire, in Portuguese), occurs in Central-Western Brazil and neighboring countries. The highest EPF prevalence has been observed in the Terena and Xavante Amerindians (over 3% and 1.4%, respectively [4]). 

The etiology and pathogenesis of pemphigus remain incompletely understood. Consequently, the therapeutic intervention is mainly based on broad systemic immunosuppression, often causing significant side effects and comorbidities. However, pemphigus onset and disease course are intimately related to environmental factors triggering the disease in individuals with a complex predisposing genetic background [5]. 

PV may be induced or triggered by drugs [6,7]. Various other potential triggers, including ultraviolet and ionizing radiation, vaccines, dietary factors, hormonal disorders, stress, and others, have been discussed [3,8,9]. In EPF, epidemiological studies indicate that continued exposure to hematophagous insect bites may be a factor triggering the disease [4,10,11]. IgG4 and IgE autoantibodies from EPF patients cross-react with a salivary protein from sand flies, suggesting that this salivary antigen could be the initial target of the immune response in EPF, which would progress to autoimmunity due to cross-reactivity and epitope spreading [12]. Conversely, we suggest that an EPF-triggering virus inoculated by a hematophagous insect in the endemic area may be the explanation for the unusual epidemiology of this autoimmune disease. 

Viral infections are known to be relevant in the context of pemphigus. Herpesviruses can trigger PV or complicate its clinical course [13]. HSV-1 (herpes simplex virus 1) and HCMV (human cytomegalovirus) are associated with PV but, apparently, not PF [14]. Serum levels of thymosin alpha I are usually normal or decreased in autoimmune diseases but increased in viral diseases [15]. Interestingly, the levels of thymosin alpha I were elevated in 73% of the 37 tested patients with EPF. These high levels were not observed in patients’ relatives, patients with other forms of pemphigus or other dermatological diseases, or healthy individuals [16].

Epidemiological and animal studies have shown that viral infection is likely to be implicated in the initiation of autoimmune diseases. In some cases, immune regulatory mechanisms for viral clearance can fail, resulting in the breakdown of self-tolerance, which culminates in immune-mediated reactions against self-antigens. Cross-reactive T cell recognition (molecular mimicry) or bystander T cell activation and epitope spreading could explain how infection can culminate in a T cell-mediated autoimmune response [17,18]. Remarkably, approximately 20% of the systemic lupus erythematosus (SLE) risk loci are putatively involved in Epstein–Barr virus (EBV) infection processes, and the risk alleles may increase the tendency toward EBV lytic switching [19].

The search for viruses associated with autoimmunity has primarily focused on known viral pathogens. A challenge is determining the possible role of viruses that do not cause overt disease in humans. Compelling evidence for an exceptional abundance and genetic diversity of viruses in many hematophagous and non-hematophagous insects has been reported [20,21,22,23,24]. 

In this context, we hypothesize that a virus inoculated by a hematophagous insect in the endemic area may trigger EPF. The putative virus may still be unknown, and the host’s response to the infection may be mild or occur unnoticed, or perhaps the symptoms of the infection precede the outbreak of EPF by some time, hindering the recognition of its association with the development of the disease. To provide insights on this topic, we conducted genetic association and mRNA expression analyses of host genes involved in the early stages of viral infections and the innate antiviral defense, showing results consistent with the involvement of a virus in EPF etiology. 

## 2. Materials and Methods

### 2.1. Patient and Control Samples

This study was performed according to the Declaration of Helsinki, approved by the Brazilian National Ethics Committee (CONEP) under the protocol number CAAE 02727412.4.0000.0096 and by the Ethics Committee of the University of Lübeck (number 08-156).

#### 2.1.1. Samples Used for the Association Analysis

DNA from 227 EPF patients and 194 controls was extracted from peripheral blood by the phenol-chloroform-isoamyl alcohol protocol [25]. The individuals in this study were unrelated and predominantly of European ancestry [26]. The control individuals were not relatives of EPF patients and had no known autoimmune disease. Patients were diagnosed by specialized dermatologists based on clinical characterization coupled with immunological tests, histopathology, and immunohistochemistry of skin biopsies. Patients and controls were recruited from the same geographical areas (Central–Western/Southeastern/Southern Brazilian hospitals) from 1984 to 2015, as previously reported by Calonga-Solís et al. [26]. The mean age was 40.9 years for patients (minimum 6, maximum 83) and 44.8 years for controls (minimum 11, maximum 86). Women were 52% in both the patient and control samples.

The sporadic PF cohort consisted of 75 patients and 150 controls whose DNA was isolated from whole blood samples using the QIAamp DNA Maxi Blood Kit (Qiagen, Hilden, Germany). Patients were diagnosed based on their clinical phenotype and positive direct immunofluorescence microscopy of a perilesional skin biopsy and/or detection of serum autoantibodies against DSG1 by ELISA (Euroimmun, Lübeck, Germany) [27]. Patients were recruited in German hospitals by the German Autoimmune Bullous Diseases Genetic Study Group, with all subjects being of European origin (predominantly of German ancestry). The mean age was 60.1 years for patients (minimum 25, maximum 88) and 60.3 years for controls (minimum 21, maximum 77). The fraction of women was 46% in the patients group and 51% in the control group. 

#### 2.1.2. Samples Used for RNA Expression Analysis 

Briefly, as previously described [28], we collected 50 mL of peripheral blood from patients with EPF and control individuals to purify different PBMC subpopulations. We used a sub-sample of that study, composed of four patients with EPF without corticosteroids treatment (3 female and 1 male), mean age was 36 years (minimum 15, maximum 54), and five controls from the endemic region (4 female and 1 male; mean age 39.6 years (minimum 22, maximum 58)). Differently from Salviano-Silva et al. [28], we used the CD4+ T lymphocyte fraction, which was obtained in a step preceding the separation of CD19+ cells, as further explained. CD14+ cells were removed with CD14 microbeads (Miltenyi Biotec, Bergisch Gladbach, Germany), and the negative fraction was then used for positive selection of CD4+ T cells using the CD4 Multisort Kit (Miltenyi Biotec, Bergisch Gladbach, Germany) and LS magnetic columns (Miltenyi Biotec, Bergisch Gladbach, Germany) according to the manufacturer’s guidelines. The purity of all nine subject’s T cell populations was assessed by flow cytometry, considering positive double staining for CD3 and CD4 markers. The mean purity for CD4+ T cells was 97% (data not shown).

### 2.2. Selection of Candidate Genes 

We initially selected genes coding for well-known viral restriction factors and other effectors of the human antiviral innate immune responses. This preliminary list included protein-coding genes whose products recognize nucleic acids, e.g., TLRs (toll-like receptors)-3, TLR-7, TLR-8 and TLR-9, IFIH1 or MDA5 (interferon induced with helicase C domain 1) or other viral structures (OAS—2’-5’-oligoadenylate synthetases enzyme family), TRIMs, IFITs (interferon-induced protein with tetratricopeptide repeats), MX1, MX2 (MX dynamin-like GTPases 1 and 2), known to take part of in signal transduction pathways (STING—stimulator of interferon response cGAMP interactor 1, IRFs—Interferon regulatory factors), or play a role in type I interferon pathway (JAK1—Janus kinase 1; TYK2—tyrosine kinase 2), or interfere with nuclear transport or viral RNAs translation (EIFs—eukaryotic translation initiation factors and NUPs—nucleoporins). Next, we added human genes found in The Gene Ontology resource by GO terms for the following biological processes: “regulation of viral entry into host cell” (GO:0046596), “negative regulation of viral-induced cytoplasmic pattern recognition receptor signaling pathway” (GO:0039532), “regulation of viral-induced cytoplasmic pattern recognition receptor signaling pathway” (GO:0039531). Finally, we included genes whose products participate in the “antiviral mechanism by IFN-stimulated genes” (R-HSA-1169410) pathway according to the Reactome database [29,30]. The complete list of 166 candidate genes is found in Table S1 and Figure S1.

### 2.3. Genotyping

Genotyping was performed by microarray hybridization (CoreExome-24 v1.1 Illumina) [31]. Among the 166 candidate genes, 20 were previously investigated in this same EPF cohort [26,32,33] and therefore were not genotyped in this study. We identified the genomic positions of each gene according to the GRCh37/hg19 human genome version, including segments of one thousand base pairs upstream and downstream from the transcription start and end sites, respectively, for the longest transcript. A total of 3063 rare single nucleotide variants (SNVs) or single nucleotide polymorphisms (SNPs) mapped to 166 candidate genes were extracted from DNA microarray data. We excluded 2254 SNVs whose minor allele frequency (MAF) was lower than 1%, 25 whose genotypic distribution deviated from those expected by Hardy–Weinberg equilibrium in the control group (*p* < 5%), and 98 in strong linkage disequilibrium (LD, r^2^ > 0.8) with one or more other variants of the dataset, remaining 686 SNPs in 147 candidate genes for the association analysis. 

SNPs associated with the EPF were genotyped in the independent cohort of patients with sporadic PF using the iPLEX platform of the MassARRAY system (Agena Bioscience, San Diego, CA, USA). The primer sequences are available in Table S2. We could not design primers for *rs1058398* (*NUP88*) due to neighboring polymorphic sites. We used the MassARRAY Typer software (v4.0) (Agena Bioscience, San Diego, CA, USA) with standard settings to call the genotypes. The genotype distribution followed the Hardy–Weinberg equilibrium in controls for all SNPs, except for *rs10160955* located within the gene *USP15*.

### 2.4. RNA-seq 

RNA was extracted from CD4+ T cells of patients and controls using mirVana^TM^ miRNA Isolation kit (Ambion, Austin, TX, USA), following the manufacturer’s protocol for total RNA extraction adapted for trizol-lysed samples. Ribosomal RNA was removed (Ribo-ZeroTM Magnetic Kit), and specific libraries were prepared using the NEB Next^®^ Ultra™ RNA Library Prep Kit. Finally, the sequencing was performed with Illumina (San Diego, CA, USA) Hi-seq platform using a paired-end 150 bp protocol. 

Reads were pseudoaligned to the human transcriptome (Ensembl version 103) using coding and non-coding genes. Quality control was performed using FastQC version 0.11.5 and quantified using Salmon version 0.11.0 with 30 bootstrap cycles. Quantification files were imported to Sleuth version 0.29.0, and we performed pairwise comparisons between EPF patients and controls from the endemic regions.

### 2.5. Statistical Analysis 

Binary logistic regression was used to perform the case-control association analyses with the PLINK software version 1.1.9 [34]. For EPF, two principal components and sex were used as co-variables. We adopted the significance limit *p* = 0.005 [35,36,37]. For validation in sporadic PF, the significance limit was *p* = 0.05. 

Global RNA-seq data analysis was performed using Sleuth package from R version 3.4.4. Furthermore, differentially expressed (DE) genes were determined based on the Wald test. The candidate genes (Table S1) were searched in this data set; those whose levels differed between patients with EPF and controls with *p* ≤ 0.001 [35,36,37] were considered DE. 

### 2.6. In Silico Analysis

The links to the used web tools can be found under “Web References”. The lists of SNPs at strong LD (r^2^ > 0.8) with the EPF-associated SNPs were obtained for the sum of the following 1000 Genomes Project European (EUR) populations: CEU—Utah residents with Northern and Western European ancestry, TSI—Tuscans in Italy, GBR—British in England and Scotland, and IBS—Iberians from Spain. The LD analyses were made with the LDproxy tool of the LDlink suite for a ±500,000 bp window. The annotation and functional impact of the 12 EPF-associated SNPs and the 450 SNPs presenting strong LD with them were performed with Ensembl, GTEx portal, UCSC, HaploReg, and SNPnexus (Tables S3 and S4). 

The physical interaction between proteins coded by genes whose SNPs were associated with EPF in this study was evaluated by STRING and GeneMANIA. We further checked the GO biological processes using FumaGWAS and performed in silico gene enrichment analysis using EnrichR with the following gene libraries: ChEA 2016 (transcription factor ChIP-Seq studies extracted from supporting material of publications), ARCHS4 (access to gene counts from HiSeq 2000 and HiSeq 2500 platforms for human and mouse experiments), and Gene Ontology 2021 for biological processes [38].

## 3. Results

We observed statistically significant (*p* < 0.005) genetic associations with variants of 11 genes and significant differential mRNA expression of another three of the tested candidate genes. 

**Figure 1:**
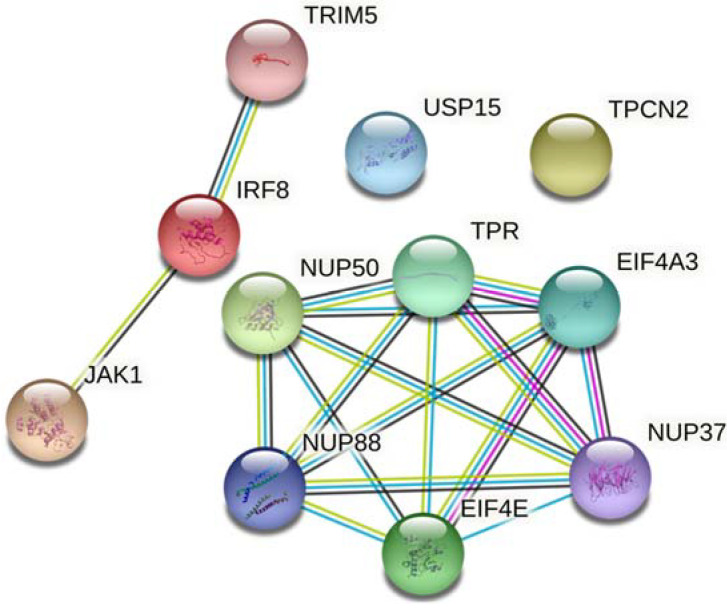
Interactions among the products of genes whose SNPs were associated with endemic pemphigus foliaceus. The interaction map was drawn using STRING (stringdb.org; accessed 2 December 2021). The edges represent protein–protein association. Blue and purple edges indicate interactions with known co-occurrence and experimental evidence, respectively. Yellow and black edges indicate textmining and co-expression evidence, respectively. Filled nodes represent proteins whose 3D structure is known or predicted, while empty nodes represent unknown 3D structures. The colors of the nodes were selected randomly.

Twelve SNPs located in the 11 genes were associated with EPF: *TRIM5* (tripartite motif containing 5), *USP15* (ubiquitin specific peptidase 15), *NUP37* (nucleoporin 37), *NUP50* (nucleoporin 50), *NUP88* (nucleoporin 88), *TPR* (translocated promoter region), *EIF4E* (eukaryotic translation initiation factor 4E), *EIF4E*3 (eukaryotic translation initiation factor 4E family member 3), *JAK1* (Janus kinase 1), *TPCN2* (or *TPC2*—two pore segment channel 2), and *IRF8* (interferon regulatory factor 8) (Table 1). Among the 12 associations, eight minor alleles were associated with increased susceptibility to EPF and four with protection. Nine SNPs are in intronic regions, two are in the 3′ UTR, and one is in an exon. The list of all SNVs analyzed is available in Table S5. We also evaluated interactions between the gene products of the associated genes (Figure 1).

The nucleoporins *NUP37, NUP50, NUP88,* and *TPR,* and the translation initiation factor *EIF4E* were all significantly enriched in GO-biological RNA transport processes: mRNA-containing ribonucleoprotein complex export, RNA, mRNA, and tRNA transport from the nucleus (GO:0071427, GO:0006405, GO:0006406, and GO:0006409, respectively) (adjusted *p*-value 7.85 × 10^−8^). Furthermore, the transcription factor E2F4 binds to the promoter of three of the genes mentioned above (*NUP37, NUP88*, *TPR*), as well as to those of *USP15, IRF8,* and *TPCN2* in lymphoblastoid GM06990 cells [39], indicating a common regulatory pathway for most associated genes.

To explore the possible causes of the detected associations, we first looked for SNPs in strong LD (r^2^ > 0.8) with the 12 EPF-associated SNPs. We found 450 SNPs. The SNP blocks included 1 to 143 SNPs, as follows: *JAK1a*—1, *JAK1b*—3, *NUP50*—5, *IRF8*—6, *TRIM5*—8, *EIF4E3*—11, *TPCN2*—16, *EIF4E*—19, *USP15*—65, *TPR*—80, *NUP37*—105, *NUP88*—143 (Table S3). 

The SNP blocks correspond to expression and/or splicing quantitative trait loci (eQTL, sQTL) in at least one tissue for all EPF-associated genes except one (*IRF8*). They are also associated with the expression of other genes, including some whose SNPs are not in LD with EPF-associated SNPs (Table S3).

For all the 77 possible transcripts of the 11 candidate genes, the predicted location and consequences of the 12 EPF-associated SNPs were: 44 in introns of coding transcripts (“coding intronic”), one exonic non-synonymous, 12 in introns of non-coding transcripts (“non-coding intronic”), one non-coding exonic, three 3′UTR, eight 5′upstream, and eight 3′downstream (Table S4). The *rs3753565* G > A SNP causes a p.Ser960Asn amino acid replacement in the major TPR isoform, which was predicted as tolerated by SIFT but possibly damaging by PolyPhen. The rs3753565*A (Asn) allele is associated with roughly 2.5 increased risk of EPF but not sporadic PF (Table 1). Some of the 450 SNPs in strong LD also have effects on the associated candidate genes: in *NUP88*, the *rs75669379* G > C causes an amino acid Arg > Gly replacement in a poorly expressed NUP88 isoform. The SNPs *rs11209*, *rs14231* and *rs1071705* C > T are coding regions’ synonymous *NUP88* variants. Moreover, the *rs739768* SNP locates very close—three nucleotides away—from a *NUP88* splice site (Table S4), which may explain the sQTL effect of the *NUP88* haplotype block (Table S3). Similarly, the *rs6591368* SNP locates three nucleotides from a *TPCN2* splice site (Table S4); however, according to GTEx, the *TPCN2* SNPs of the haplotype block are not sQTLs (Table S3). Transcripts of *NUP88*, *NUP50, EIF4E*, *IRF8*, and *USP15* present SNPs in the 3′UTR, whereas only *NUP88* and *TRIM5* have SNPs in the 5′UTR (Table S4).

The annotation of all the 462 SNPs revealed another 10 protein-coding and five ncRNA genes (and four pseudogenes) for which gene/protein consequences may occur (Table S4): among 2750 possible consequences for the transcripts of the complete gene set, 1656 (60%) were for protein-coding genes (1620 intronic, 41 coding synonymous, 15 coding non-synonymous), 670 (24.3%) were for protein-coding genes (617 intronic, 53 exonic), 185 (6.7%) were 5′upstream, 189 (6.9%) 3′ were downstream, and 30 (1.1%) were in untranslated regions (UTR) of the transcripts, of which 26 were in 3′UTR) (Table S4). 

Nine of the 12 EPF-associated SNPs were evaluated in the sporadic PF cohort. The *NUP88 rs1058398* could not be genotyped by iPlex due to polymorphic sites neighboring this SNP, which precluded primer designing, while the frequency of the minor alleles of *EIF4E rs6834230* and *TPCN2 rs4930263* SNPs was zero in that population. Only two associations—rs1447904*C of *EIF4E3* and rs10160955*C of *USP15*—were validated for sporadic PF (Table 1). However, the *USP15* SNP genotype frequencies deviated from those expected under Hardy–Weinberg equilibrium in the control group of sporadic PF. These results suggest differences in the early stages of EPF and sporadic PF pathogenesis. 

Differential gene expression analysis revealed that the following genes were significantly overexpressed in CD4+ T lymphocytes obtained from EPF patients: *CCL5* (C-C motif chemokine ligand 5), *P4HB* (prolyl 4-hydroxylase subunit beta), and *APOBEC3G* (apolipoprotein B mRNA editing enzyme catalytic subunit 3G) (Table 2). *APOBEC3G* and *CCL5* are negative regulators of viral replication (GO:0045071) and are co-expressed with several transcription factors: *BATF, PRDM1* (also known as *BLIMP1*)*, ZBTB32, ID2, CREM, STAT4, HOPX, ZNF80,* and *ZNF600,* all of which (except for the last three, which are poorly known) act on T cell differentiation within the antiviral response (adjusted *p* = 0.0067). We found that four of the seven differentially expressed (DE) genes identified in CD4+ T lymphocytes of EPF patients from a former study of our group [40]—*IFITM3, OAS1, APOBEC3A,* and *OASL*—are co-expressed with the antiviral transcription factors *BATF2, STAT2, ETV7* and *IRF7* (adjusted *p* = 0.00025) [41,42,43]. The list of all genes whose RNA levels were analyzed is available in Table S6). Borderline significance suggestive of differential expression (0.005 < *p* < 0.05) was observed for other 14 genes, including the genetically associated *NUP37* and *NUP50,* which were overexpressed in patients’ CD4+ T cells (Table S6). 

Overall, these results are consistent with the involvement of viruses in the etiology of EPF. 

## 4. Discussion

The limited geographic distribution and high incidence of EPF (*fogo selvagem*) in Brazil indicate a yet undiscovered environmental factor triggering the disease in genetically susceptible individuals living in the endemic area [5]. Evidence points to the involvement of some substance inoculated by a hematophagous insect, notably a black fly of the genus *Simulium* or the *Lutzomyia longipalpis* sandfly [10,11,12,44]. The triggering factor could be a salivary protein or a virus. The possibility that salivary proteins of insects could induce EPF has been considered to some extent [11,12,45,46], but the hypothesis of a viral trigger has not received much attention. However, viruses have been associated with several autoimmune diseases, and autoimmune manifestations may occur in viral infections [47,48]. 

We hypothesized that a virus could be the primary trigger of EPF. As an indirect approach to testing this hypothesis, we searched for variants in genes involved in the antiviral innate immune responses in a case-control study of EPF and compared the mRNA expression of the same set of candidate genes in CD4+ T cells of patients with EPF and controls living in the endemic regions. The rationale for this approach was that genetic associations and differential gene expression indicate variability in the extent or effectiveness of the antiviral response, which, in turn, could permit the establishment of viral infection in susceptible individuals. The cross-reactivity of antigens of the putative virus with the targets of autoimmunity in EPF (primarily DSG1) and epitope spreading would initiate the development of the pathologic autoimmunity characteristic of EPF. We found significant genetic associations for 11 and differential expression (DE) of three genes involved in the early stages of viral infections in EPF. These results corroborate the hypothesis of a viral trigger in this endemic autoimmune disease. 

The EPF-associated variants were then analyzed in an independent cohort of sporadic PF and controls. Only two—*USP15* and *EIF4E3*—of the eight genes whose variants were informative for association analysis in sporadic pemphigus foliaceus (PF) were associated in that cohort, which is in line with our hypothesis of a shared viral trigger for EPF and more diverse, rare environmental triggers for sporadic PF. 

Many genes whose products are essential for antiviral responses are also involved in autoimmune diseases. Although, especially the *TRIM5*, *TPCN2, EIF4E*, *EIF4E3*, *APOBEC3G, P4HB*, *NUP37, NUP50, NUP88*, and *TPR* genes are crucial in antiviral host responses but show no or at best scarce evidence of involvement in other autoimmune diseases. 

We observed a genetic association of *TRIM5* with EPF. The TRIM5α isoform is a pattern recognition receptor in innate immunity [49] and an intrinsic restriction factor for many retroviruses [50] and tick-borne flaviviruses but not several mosquito-borne flaviviruses [51]. TRIM5α induces premature uncoating of the viral genome, blocks reverse transcription, and promotes an antiviral host state [52]. In rhesus monkeys, but not humans, TRIM5α confers resistance to HIV-1 [53]. TRIM5α also controls the endogenous mobile genetic LINE-1 elements [49]. A *TRIM5* SNP was associated with increased odds of rapid, early, and sustained virological response after interferon-based therapy in patients with chronic hepatitis C or HIV/HCV coinfection [54,55]. Viruses and HERVs may play a role in the etiology of multiple sclerosis (MS) [56,57,58]. A haplotype block of eight *TRIM5* SNPs within the 5′UTR of the transcript that codes for the TRIM5α isoform (Ensembl accessed 26 November 2021) was associated with lower susceptibility to MS [59]; however, the EPF and MS haplotypes are not associated one with each other (r^2^ ~ 0.04). 

The *APOBEC3G* mRNA expression was increased in CD4+ T cells of patients with EPF compared to control individuals. Genetic associations with *APOBEC3G* gene variants were not observed in EPF or any other autoimmune disease. Viruses can antagonize APOBEC function through viral proteins and ncRNAs [60]. However, the risk of HIV-1 infection, viral load, or the course of HIV-1 disease were associated with *APOBEC3G* polymorphisms [61]. Human A3G exhibits potent antiviral activity against viral infectivity factor (Vif)-deficient HIV-1 [62] and other viruses, such as HBV, HCV, and HTLV [60,61]. Together with other APOBEC enzymes, A3G provides a barrier for SIV (simian immunodeficiency virus) infection in humans [63]. The APOBEC3G (A3G) enzyme is a member of the APOBEC3 (A3) family of intrinsic viral restriction factors [64]. A3G inhibits retrovirus replication and the retrotransposition of HERVs [65]. The A3G and other A3 RNAs were overexpressed in the serum of SLE patients compared to healthy controls [66] and in the minor salivary glands of patients with Sjogren’s Syndrome lymphoma [67].

There are two human TPC genes. The *TPCN2* (or *TPC2*) gene is ubiquitously expressed [68,69]. We observed an association between increased EPF susceptibility and *TPCN2 rs4930263 AA* and *AC* genotypes. TPCN2 participates in several endolysosomal processes, such as trafficking, exocytosis, autophagy, and lysosomal cation/pH homeostasis [69]. Most enveloped viruses, including filoviruses (e.g., the Ebola virus) and coronaviruses, access the host cells via endocytosis and require TPCN2 to enter the cytoplasm [70,71,72]. 

We found genetic associations between EPF and variants within *EIF4E* and *EIF4E3*. *EIF4E* was reported as DE in PV [73]. Involvement of these genes in other autoimmune diseases has not yet been reported, but numerous studies pointed to their involvement in viral infections. The host cells’ translation machinery is required for viral replication and virion synthesis and can be regulated by viral or host factors to favor or restrict viral processes [74]. The heterotrimeric eIF4F complex is essential for cap-dependent protein synthesis [74,75]. It facilitates the interaction between the ribosome and the mRNA through binding to the mRNA 5′-cap structure (by eIF4E), RNA unwinding (eIF4A), and interaction with eIF3 (eIF4G), which is bound to the small ribosomal subunit. The silencing of the eIF4F complex by the knockdown and knockout of eiF4E and eIF4G suppresses the levels of IRF1 and IRF7, compromising their ability to inhibit rotavirus replication [74]. In cells exposed to stress, such as low oxygen conditions, the canonical eIF4F is replaced by alternative eIF4Fs which use eIF4E (such as EIF4E3) and eIF4G homologs [76,77]. Viral infections may induce such stress conditions. Several DNA and RNA viruses exhibit eIF4E-dependent translation and replication [78,79,80], while eIF4E-independent replication occurs for other viruses [80,81,82]. Moreover, the VSV and other eIF4E-independent viruses may disrupt the eIF4F complex, inhibiting host protein synthesis [83,84]. It seems that DENV uses cap-dependent translation at the early stages of infection and then switches to cap-independent translation as seen by downregulation of several eIF4F components, including eIF4E, and inhibition of translation [85].

The nuclear pore complex (NPC) is a multiprotein structure composed of ≈30 different nucleoproteins, the nucleoporins (NUPs). It regulates molecular transport across the nuclear envelope (NE) and is involved in gene expression. In our study, variants of the NPC genes *NUP37, NUP50, NUP88,* and *TPR* were associated with differential risk of EPF. The NPC proteins may be targets in autoimmune diseases [86,87,88]. However, genetic variants or altered expression have not yet been implicated in autoimmune diseases, except for *NUP88* in two GWAS of rheumatoid arthritis (RA) [89,90]. The SNPs associated with EPF and RA do not present high LD (*rs1058398* and *rs72634030*, respectively; LDlink accessed 28 October 2021). In contrast with the scarce reports for autoimmune diseases, NPC plays a crucial role in viral replication. Many viruses hijack the NPC to regulate the nucleocytoplasmic trafficking of viral and host macromolecules, promoting viral replication and affecting host cell pathways to evade antiviral responses [91]. Proteases of flaviviruses (such as DENV, ZIKV, and YFV) and picornaviruses degrade nucleoporins to inhibit the nuclear import/export of host molecules involved in the immune response [92,93]. TPR is one of the NPC proteins degraded by a ZIKV protease [93] and may promote HIV infection by ensuring that the chromatin environment near the nuclear pore is active, with implications for the preferential integration of HIV into actively transcribed genes [94]. On the other hand, HIV-1 infection can be inhibited via a mechanism that involves targeted repression of *NUP50* [95]. Furthermore, *NUP37* is among the 22 overexpressed genes in EBV-transformed lymphocytes and may be one of the key regulatory genes of lymphocyte transformation induced by EBV [96].

The *USP15 rs10160955 CC* genotype is associated with decreased susceptibility to EPF. The USP15 is one of the ≈70 ubiquitin-specific proteases (USPs), which is a family involved in several signaling pathways [97]. USP15 regulates the TRIM25- and DDX58 (RIG-I)-mediated antiviral immune response [98,99]. The human papillomavirus (HPV) E6 oncoprotein antagonizes the activation of DDX58 by targeting TRIM25 and USP15 [100]. Conversely, the interaction between USP15 and the HIV-1 Gag and Nef (necessary for AIDS pathogenicity) inhibits HIV-1 replication. The interaction between Nef and USP15 leads to reciprocal decay of the proteins, and the balance of USP15/Nef interplay underlines the dynamic competition between the virus and the infected host cells [101]. Further, USP15 is a host factor for hepatitis C virus (HCV) propagation [102] and participates in the differentiation of Th17 cells [103], which are involved in immunity against pathogens and autoimmune diseases, including MS and psoriasis [104,105]. 

The redox-regulated protein disulfide-isomerase (PDI) P4HB (PDIA1) RNA was significantly overexpressed in CD4+ T cells of patients with EPF compared to controls. P4HB is one of the over 20 members of the PDI family. PDIs play a fundamental role in the protein folding of numerous viruses, including IAV, HCV, DENV, HSV-1, HIV, and coronaviruses [106]. Analysis of the protein profile of HSV-1 infected primary corneal epithelial cells indicated P4HB as one of the critical proteins in the antiviral response [107]. Conversely, the involvement of P4HB in autoimmunity was shown to result from cross-reactivity with microbial antigens. The cross-reactivity of P4HB with anti-DENV nonstructural protein 1 (NS1) antibodies may play a role in DENV infection-induced autoimmunity [108]. In some SLE patients, antibodies against the HU1 bacterial peptide recognized P4HB as an autoantigen on the membrane of renal cells, inducing lupus nephritis [109]. Thus far, polymorphisms in *P4HB* have not been related to complex diseases. 

The CCL5 (RANTES) chemokine is secreted mainly by T cells and is involved in immunoregulatory and inflammatory processes. We observed an increased *CCL5* mRNA expression in CD4+ T cells of patients with EPF. During MS relapse and in other inflammatory neurological diseases, increased CCL5 protein levels were observed in patients’ cerebrospinal fluid and serum. However, CCL5 levels were lower in stable relapsing-remitting MS and progressive MS than in MS relapse and below the detection limit in patients with the non-inflammatory neurological disease [110]. The overexpression of CCL5 was also reported in several autoimmune thyroid disorders [111]. CCL5 is one of the major HIV-suppressive factors produced by CD8+ T cells [112,113], and high levels of CC-chemokines, including CCL5, suppress the replication of IAV-infected cells [114]. Moreover, during acute HCV infection, the increased expression of CCL5 is crucial for the induction of Th1 responses and the control of HCV infection and liver disease [115]. Genotypes or haplotypes associated with low CCL5 levels increased the susceptibility to severe enterovirus 71 infections [116] and tuberculosis [117]. 

Polymorphisms within or near the *IRF8* gene were associated with susceptibility to EPF and other diseases such as MS, SLE, and SSc [118,119,120,121], as well as Behçet’s disease but not Vogt–Koyanagi–Harada syndrome [122]. The SNPs associated with MS, SLE, SSc, and Behçet’s disease are not in LD with the EPF-associated *rs1044873* SNP (LDlink accessed 18 October 2021). To identify molecular mechanisms shared between pemphigus and SLE, Sezin et al. found 3280 genes co-expressed in CD4+ T cells of pemphigus and patients with SLE [123]. The “type I interferon signaling pathway” and “defense response to virus” pathways were enriched in one cluster of co-expressed genes significantly overexpressed for both diseases. By associating the co-expressed genes with GWAS results for pemphigus and SLE, *IRF8* and *STAT1* were characterized as the key regulatory genes [123].

The JAK/STAT signal transduction pathway is involved in many cellular processes and is critical for resisting infection and maintaining immune tolerance. The Janus kinases (JAK1, JAK2, JAK3, TYK2) are located intracellularly and downstream of interferon and cytokine receptors [124,125]. *JAK1* polymorphisms were associated with variable risk for EPF and autoimmune thyroid disease [126,127], psoriasis [128,129], Behçet’s disease [130], and Vogt–Koyanagi–Harada syndrome [131]. Autoimmune traits were also associated with a *JAK1* polymorphism in a GWAS of a large European cohort [132]. The expression of *JAK1* was altered in diverse viral diseases and/or viruses that targeted the JAK1 protein to modulate the host’s immune response [133,134,135,136,137,138]. 

We searched for the possible reasons for the genetic associations observed employing in silico analyses. As usual for association analyses with tag SNPs, the causal variants may be others in strong LD with the disease-associated SNPs. The functional annotation of all SNPs—the 12 EPF-associated plus 450 in strong LD with them—revealed that apart from the 11 associated candidate genes, part of the SNPs was shared with 10 (partially) overlapped protein-coding and five non-coding genes (besides four pseudogenes). We looked for the functions of these genes and did not find any evidence of their involvement in innate immune response, antiviral defense, or autoimmune disease, turning them into minor candidates in our study. We found that the 12 EPF-associated SNPs/haplotype blocks are eQTL or sQTL for all but one (*IRF8*) candidate genes in at least one tissue. This result suggests the effect on expression levels of the 11 candidate genes as the more likely reason for the genetic associations. A *NUP88* SNP is only three nucleotides away from a *NUP88* splice site and may be responsible for the sQTL effect of the *NUP88* haplotype. Some transcripts of *NUP50*, *NUP88*, *EIF4E*, *IRF8*, and *USP15* bear SNPs in the 3′UTR, and *NUP88* and *TRIM5* have SNPs in the 5′UTR. Moreover, the EPF-associated *TPR* SNP causes an amino acid replacement in the major TPR isoform, which, besides the eQTL effect of the *TPR* haplotype block, might contribute to increased susceptibility to EPF. 

Further support of a viral trigger in EPF is the protective effect of natural killer (NK) cells in EPF. The killer-cell immunoglobulin-like receptor (*KIR*) genes are responsible for transducing signals that control NK-mediated cytotoxicity against viral infections. The activating *KIR* genes are often reported to increase the risk of autoimmune diseases and reduce the risk of infectious diseases [139], which is consistent with the impact of NK cell activation on viral clearance [140]. Interestingly, increased numbers of activating genes and higher ratios of activating/inhibitory signals have been previously associated with protection against EPF in a former study of our group [141], which is the opposite pattern that we would expect for autoimmune diseases. Later, our group also demonstrated that reduced expression of the inhibitory KIR3DL2 protects against EPF [142], which is further evidence that NK cell activation is unusually protective for this autoimmune disease.

Among the markers of 20 additional candidate genes involved in B cell development and antibody production tested for associations in a previous work of our group (Table S1), only the rs2070729*C allele of *IRF1* (interferon regulatory factor 1) was associated with increased susceptibility to EPF [26]. The IRF1 has a broad range of functions in maintaining homeostasis and protecting the host from invading pathogens [143].

In a previous EPF genome-wide expression study (GWES), several genes involved in the early stages of viral infection were overexpressed in patients with the generalized form of EPF compared to healthy individuals, including *IFITM3, APOBEC3A, APOBEC3G, OAS1, OASL, SERINC3,* and *RPLP2* [40]. Interestingly, *IFITM3, APOBEC3A, OAS1, OASL, RPLP2,* and *CCL5* are activated by IRF1 [144], and RPLP2 is required for flaviviral translation [145]. When subgroups of patients selected according to disease severity or treatment were compared, genes involved in the innate antiviral response were not DE [40]. Compared with EPF, fewer DE genes related to early stages of viral infections were observed in PV GWESs. Among all the genes associated or DE in our EPF study, only *EIF4E* and *USP15* were overexpressed in patients with PV compared with HLA-matched controls [73], while *NUP50* was downregulated in keratinocytes incubated in vitro with PV serum compared with normal serum [146]. None of the other genes associated or DE in EPF were DE in two other PV GWESs [147,148]. 

Interestingly, the genetic associations with EPF were not observed in the sample of sporadic PF, with the only exception of *USP15* and a borderline association with *EIF4E3*, but *NUP88*, *EIF4E,* and *TPCN2* could not be analyzed in this sample. The EPF endemicity indicates a unique triggering environmental factor in the high-prevalence areas. This peculiarity is not a characteristic of the other form of PF, which occurs sporadically across the globe and may be triggered by different environmental factors and probably other viruses. The heterogeneity of the sporadic PF triggers could hamper the detection of associations with variants of genes implicated in viral host responses, also explaining the distinct set of associations observed only in EPF.

Several limitations of our study must be considered. The genes analyzed undoubtedly do not cover all candidates. For example, we did not consider later stages of the viral cycle, such as viral latency or budding. Our gene expression analysis was limited to CD4+ T cells, and the number of analyzed individuals was small. Some of our results could also be explained by other inflammatory stimuli that lead to IFN signaling. Although we discuss the function of the relevant gene products and the consequences of their variability in viral infections and autoimmune diseases, the causes of the associations could not be addressed and should be the subject of future work. Nevertheless, our results provide evidence that a virus may trigger EPF, presumably a still unnoticed flavivirus. We expect this work will encourage further research, leading to discoveries about the environmental trigger of *fogo selvagem*.

## 5. Conclusions

We provide evidence that a virus triggers endemic pemphigus foliaceus (*fogo selvagem*). Overall, host genetics points to a flavivirus or retrovirus, but given the longstanding evidence of a hematophagous insect’s involvement in the etiology of EPF, flaviviruses are the primary candidates. The EPF endemic region is more restricted than the geographical distribution of the candidate insects, which is difficult to reconcile with an insect’s saliva protein as the primary trigger of the disease. Conversely, the interplay of an insect and an animal reservoir of the putative virus could explain the limited geographical distribution of the disease. We hope that our findings will lay the foundation for identifying the presumed virus and vectors and the molecular mechanisms that underlie the tolerance breakdown in EPF.

## Figures and Tables

**Table 1:** Genetic variants associated with endemic pemphigus foliaceus and association analysis of the same variants in sporadic pemphigus foliaceus.

		Endemic Pemphigus Foliaceus								Sporadic Pemphigus Foliaceus							
GENE	SNP	MAF (%)		MODEL	CTR	PAT	OR	95% CI	*p*	MAF (%)		MODEL	CTR	PAT	OR	95% CI	*p*
		CTR	PAT							CTR	PAT						
*TRIM5*	*rs4992800*	7.25	14.64	**add**	**28/360**	**65/379**	**2.21**	**[1.37–3.56]**	**0.0012**	2.40	3.33	add	7/285	5/145	1.34	[0.45–4.0]	0.5941
11p15.4	*a > C*			rec	1/193	5/217	4.53	[0.52–39.17]	0.17			rec	1/145	0/75	NA	NA	NA
	Intron 1			**dom**	**27/167**	**60/162**	**2.30**	**[1.38–3.86]**	**0.0014**			dom	6/140	5/70	1.67	[0.49–5.65]	0.4122
*USP15*	*rs10160955*	22.16	13.66	**add**	**86/302**	**62/392**	**0.56**	**[0.39–0.81]**	**0.0017**	15.69	25.33	add	43/231	38/112	1.74	[1.07–2.79]	0.0234
12q14.1	*T > c*			rec	12/182	4/223	0.27	[0.08–0.84]	0.0246			rec	8/129	3/72	0.67	[0.17–2.61]	0.566
	Intron 20			dom	74/120	58/169	0.17	[0.00–17.43]	0.4496			**dom**	**35/102**	**35/40**	**2.55**	**[1.40–4.62]**	**0.0020**
*NUP37*	*rs11111162*	19.37	26.89	add	74/308	121/329	1.44	[1.03–2.00]	0.0292	23.08	19.33	add	66/220	29/121	0.80	[0.49–1.30]	0.3671
12q23.2	*A > g*			**rec**	**4/187**	**22/203**	**4.80**	**[1.62–14.3]**	**0.0047**			rec	7/136	3/72	0.81	[0.20–3.22]	0.7645
	Intron 4			dom	70/121	99/126	1.28	[0.86–1.91]	0.2257			dom	59/84	26/49	0.75	[0.42–1.35]	0.3437
*NUP50*	*rs2138156*	30.67	41.85	**add**	**119/269**	**190/264**	**1.56**	**[1.18–2.07]**	**0.0018**	30.14	38.00	add	88/204	57/93	1.40	[0.93–2.10]	0.1047
22q13.31	*C > t*			**rec**	**19/175**	**45/182**	**2.29**	**[1.28–4.07]**	**0.0049**			rec	13/133	13/62	2.14	[0.94–4.90]	0.0700
	Intron 3			dom	100/94	145/82	1.62	[1.09–2.41]	0.0158			dom	75/71	44/31	1.34	[0.76–2.36	0.3035
*NUP88*	*rs1058398*	45.36	38.55	add	176/212	175/279	0.72	[0.54–0.96]	0.0259	-	-	-	-	-	-	-	-
17p13.2	*A > g*			rec	33/161	36/191	0.87	[0.52–1.48]	0.0517			-	-	-	-	-	-
	3′UTR			**dom**	**143/51**	**139/88**	**0.54**	**[0.35–0.83]**	**0.0046**			**-**	**-**	**-**	**-**	**-**	**-**
*TPR*	*rs3753565*	4.40	9.47	**add**	**17/369**	**43/411**	**2.43**	**[1.35–4.4]**	**0.0031**	13.1	15.33	add	38/252	23/127	1.17	[0.70–1.98]	0.5491
*1q31.1*	*G > a* Ser > Asn			rec	1/192	2/225	1.33	[0.11–15.94]	0.8216			rec	4/141	4/71	1.99	[0.48–8.17]	0.3419
	Exon 22			**dom**	**16/177**	**41/186**	**2.68**	**[1.44–4.99]**	**0.0018**			dom	34/111	19/56	1.11	[0.58–2.11]	0.7567
*EIF4E*	*rs6834230*	6.96	3.56	**add**	**27/361**	**16/434**	**0.37**	**[0.18–0.73]**	**0.0045**	0	0	add	NA	NA	NA	NA	NA
4q23	*C > t*			rec	1/193	0/225	-	-	-			rec	NA	NA	NA	NA	NA
	Intron 1			dom	26/168	16/209	0.37	[0.18–0.75]	0.0057			dom	NA	NA	NA	NA	NA
*EIF4E3*	*rs1447904*	27.32	36.73	add	106/282	166/286	1.49	[1.10–2.00]	0.0094	26.41	35.33	add	75/209	53/97	1.51	[0.99–2.32]	0.0573
3p13	*T > c*			rec	17/177	29/197	1.40	[0.73–2.66]	0.308			**rec**	**9/133**	**11/64**	**2.54**	**[1.00–6.44]**	**0.0495**
	Intron 2			**dom**	**89/105**	**137/89**	**1.76**	**[1.19–2.60]**	**0.0046**			dom	66/76	42/33	1.47	[0.83–2.57]	0.183
*JAK1*	*rs310199*	41.75	49.12	add	162/266	231/223	1.39	[1.05–1.83]	0.0205	28.67	27.7	add	82/204	41/107	0.95	[0.60–1.67]	0.8229
1p31.3	*A > g*			rec	39/155	56/171	1.23	[0.77–1.97]	0.3881			rec	10/133	3/71	0.56	[0.15–2.10]	0.3929
	Intron 2			**dom**	**123/71**	**175/52**	**1.86**	**[1.21–2.86]**	**0.0046**			dom	39/155	56/171	1.04	[0.59–1.82]	0.8887
	*rs310202*	30.05	39.6	add	116/270	179/273	1.48	[1.10–2.0]	0.0102	20.63	20.67	add	59/227	31/119	1.00	[0.60–1.67]	0.9923
	*A > g*			rec	19/174	31/195	1.35	[0.73–2.50]	0.3382			rec	4/139	2/73	0.95	[0.17–5.32]	0.9554
	Intron 2			**dom**	**97/96**	**148/78**	**1.781**	**[1.20–2.65]**	**0.0045**			dom	55/88	29/46	1.00	[0.57–1.79]	0.9764
*TPCN2*	*rs4930263*	2.85	8.62	**add**	**11/375**	**39/413**	**2.76**	**[1.41–5.40]**	**0.0031**	0	0.68	add	0/276	1/145	NA	NA	NA
11q13.3	*a > C*			rec	1/192	4/222	3.01	[0.33–27.5]	0.328			rec	0/138	0/73	NA	NA	NA
	Intron 16			**dom**	**10/183**	**35/191**	**3.20**	**[1.53–6.69]**	**0.0019**			dom	0/138	1/72	NA	NA	NA
*IRF8*	*rs1044873*	42.27	36.5	add	164/224	165/287	0.76	[0.57–1.02]	0.0644	41.9	38.5	add	119/165	57/91	0.87	[0.58–1.30]	0.5014
16q24.1	*C > t*			**rec**	**39/155**	**23/203**	**0.42**	**[0.24–0.73]**	**0.0024**			rec	26/116	11/63	0.78	[0.33–0.68]	0.5243
	3′ UTR			dom	125/69	142/84	0.93	[0.62–1.39]	0.7186			dom	93/49	46/28	0.87	[0.48–1.55]	0.6278

Logistic regression association tests were performed with allele frequencies (“add”—additive model), frequency of homozygotes for the minor allele (“rec”—recessive model), and summed frequency of heterozygotes and homozygotes for the minor allele (“dom”—dominant model). The minor alleles in our population are the reference for the association analyses and are given in lowercase. Ser > Asn, serine > asparagine amino acid replacement; In bold: significant associations (*p* < 0.005) and suggestive associations (0.005 < *p* < 0.05); SNP, single nucleotide polymorphism; MAF, minor allele frequency in our population; CONTR, controls; PAT, patients; Model, association tests; OR, odds ratio; CI, confidence interval; PF, pemphigus foliaceus. *TRIM5*, tripartite motif containing 5; *USP15*, ubiquitin specific peptidase 15; *NUP37*, nucleoporin 37; *NUP50*, nucleoporin 50; *NUP88* nucleoporin 88; *TPR*, translocated promoter region; *EIF4E*, eukaryotic translation initiation factor 4E; *EIF4E3*, eukaryotic translation initiation factor 4E family member 3; *JAK1*, Janus kinase 1; *TPCN2*, two pore segment channel 2; *IRF8* interferon regulatory factor 8. The genotypes of all SNPs were in Hardy Weinberg equilibrium, except for *rs10160955* (*USP15*) in the sporadic PF control sample (*p* = 0.006).

**Table 2:** Genes differentially expressed (DE) at the mRNA level in endemic pemphigus foliaceus patients compared to controls.

Genes	*p*	Fold Change
*CCL5*	3.885 × 10^−06^	1.4362
*P4HB*	3.730 × 10^−05^	0.4185
*APOBEC3G*	4.212 × 10^−05^	0.5244

*CCL5* (C-C motif chemokine ligand 5), *P4HB* (prolyl 4-hydroxylase subunit beta), *APOBEC3G* (apolipoprotein B mRNA editing enzyme catalytic subunit 3G).

## Data Availability

Not applicable.

## Supplementary Materials

The following supporting information can be downloaded at: https://www.mdpi.com/article/10.3390/v14050879/s1, Figure S1: GO biological processes of molecules included in this study; Table S1: Genomic positions of genes investigated; Table S2: Primer sequences for genotyping with the massARRAY iPLEX Platform; Table S3: Annotation of EPF-associated SNPs (bold) and the 450 SNPs in high LD (r^2^ > 0.8 in 1000G EUR excluding FIN) with the EPF-associated SNPs; Table S4: Predicted Gene/Protein Consequences of the 12 EPF-associated SNPs (in bold) and the 450 SNPs at high LD (r^2^ > 0.8) with them (Ensembl by SNPnexus); Table S5: Results of the EPF-association analysis for all the selected single nucleotide variants (SNVs); Table S6: Results of the RNA expression analysys in CD4+ T cells from EPF patients compared to controls from the endemic region.
